# Editorial: Integrative physiological approaches to understand high altitude adaptation

**DOI:** 10.3389/fphys.2024.1487290

**Published:** 2024-09-06

**Authors:** Esteban Ortiz-Prado, Niroj Kumar Sethy, Jorge Vasconez-Gonzalez, Juan S. Izquierdo-Condoy

**Affiliations:** ^1^ One Health Research Group, Universidad de Las Americas, Quito, Ecuador; ^2^ Defence Institute of Physiology and Allied Sciences (DRDO), New Delhi, India

**Keywords:** high altitude (low air pressure), adaptation, physiology, new insights, editorial/personal viewpoint

The Research Topic “*Integrative physiological approaches to understand high altitude adaptation*” showcases pioneering research into the physiological mechanisms that enable humans and other organisms to thrive in high-altitude environments. Characterized by low oxygen levels, these environments demand complex adaptations across multiple physiological systems (Xi et al., 2016). This Research Topic of articles integrates perspectives from molecular biology, physiology, and evolutionary biology, offering a comprehensive understanding of high-altitude adaptation.

High-altitude environments rank among the most extreme habitats on Earth, demanding significant physiological adjustments for survival. Globally, at least 5.7% of the population lives above 1,500 m, with millions chronically exposed to these conditions (Tremblay and Ainslie, 2021). Over the past few years, research has increasingly focused on integrative approaches to study these adaptations, emphasizing the importance of considering multiple physiological systems and their interactions. Studies have ranged from analyses of lung function and cardiovascular risk to anthropometric changes and even the effects on optimism (Izquierdo-Condoy et al., 2022; Ortiz-Prado et al., 2022a; Ortiz-Prado et al., 2021; Ortiz-Prado et al., 2022b; Ortiz-Prado et al., 2022c). The studies featured in this Research Topic, with over 14,000 views and downloads, reflect the growing recognition of the complexity of high-altitude adaptation.

The Research Topic includes five manuscripts, each contributing valuable insights into different aspects of high-altitude physiology. The diversity of approaches underscores the multifaceted nature of adaptation in these environments.

Moya et al. explore physiological differences between Tibetans and Han Chinese living at intermediate altitudes, discovering that Tibetan males exhibit lower hemoglobin concentrations and a blunted heart rate response to hypoxia compared to Han Chinese. This suggests that some high-altitude adaptations are retained even at moderate elevations, likely due to genetic factors. For their part, Ding et al. investigate the effects of acute hypoxia on ovarian function, revealing that high-altitude environments may impact reproductive health by disrupting circadian rhythms, a novel area in high-altitude physiology.

Wang et al.’s review addresses the impact of high altitude on renal physiology and kidney disease, a topic that has received less attention than cardiovascular and respiratory adaptations. Their work underscores the need for further research on how chronic hypoxia affects renal function and contributes to kidney disease. Meanwhile, Zila-Velasque et al. provide a bibliometric analysis of research on adaptation and altitude sickness over the past 40 years, identifying key trends and collaborative networks, and highlighting areas for further investigation.

Finally, Gao et al. examine the incidence and risk factors of severe acute high-altitude illness in individuals newly exposed to high altitudes, offering insights with implications for prevention and treatment strategies.

Collectively, these articles emphasize the importance of integrative approaches in understanding the complex adaptations necessary for survival at high altitudes. By considering interactions among various physiological systems, these studies offer a more complete understanding of how organisms adapt to hypoxia. The insights gained from this research have broad implications for high-altitude biology and related fields such as medicine, evolutionary biology, and environmental science.

As we conclude this Research Topic, we extend our gratitude to all the contributing authors for their work, which has significantly advanced our understanding of high-altitude adaptation. We also thank the reviewers for their rigorous evaluations, ensuring the high quality of the published articles. We hope this Research Topic will inspire further research in the fascinating field of high-altitude physiology and serve as a valuable resource for researchers and practitioners alike.
